# Digital Medicine Community Perspectives and Challenges: Survey Study

**DOI:** 10.2196/24570

**Published:** 2021-02-03

**Authors:** Brinnae Bent, Ida Sim, Jessilyn P Dunn

**Affiliations:** 1 Department of Biomedical Engineering Duke University Durham, NC United States; 2 Department of Medicine University of California, San Francisco San Francisco, CA United States; 3 Department of Biostatistics & Bioinformatics Duke University Medical Center Durham, NC United States

**Keywords:** digital medicine, digital health, interoperability, mHealth, wearables, sensors

## Abstract

**Background:**

The field of digital medicine has seen rapid growth over the past decade. With this unfettered growth, challenges surrounding interoperability have emerged as a critical barrier to translating digital medicine into practice. In order to understand how to mitigate challenges in digital medicine research and practice, this community must understand the landscape of digital medicine professionals, which digital medicine tools are being used and how, and user perspectives on current challenges in the field of digital medicine.

**Objective:**

The primary objective of this study is to provide information to the digital medicine community that is working to establish frameworks and best practices for interoperability in digital medicine. We sought to learn about the background of digital medicine professionals and determine which sensors and file types are being used most commonly in digital medicine research. We also sought to understand perspectives on digital medicine interoperability.

**Methods:**

We used a web-based survey to query a total of 56 digital medicine professionals from May 1, 2020, to July 10, 2020, on their educational and work experience, the sensors, file types, and toolkits they use professionally, and their perspectives on interoperability in digital medicine.

**Results:**

We determined that the digital medicine community comes from diverse educational backgrounds and uses a variety of sensors and file types. Sensors measuring physical activity and the cardiovascular system are the most frequently used, and smartphones continue to be the dominant source of digital health information collection in the digital medicine community. We show that there is not a general consensus on file types in digital medicine, and data are currently handled in multiple ways. There is consensus that interoperability is a critical impediment in digital medicine, with 93% (52) of survey respondents in agreement. However, only 36% (20) of respondents currently use tools for interoperability in digital medicine. We identified three key interoperability needs to be met: integration with electronic health records, implementation of standard data schemas, and standard and verifiable methods for digital medicine research. We show that digital medicine professionals are eager to adopt new tools to solve interoperability problems, and we suggest tools to support digital medicine interoperability.

**Conclusions:**

Understanding the digital medicine community, the sensors and file types they use, and their perspectives on interoperability will enable the development and implementation of solutions that fill critical interoperability gaps in digital medicine. The challenges to interoperability outlined by this study will drive the next steps in creating an interoperable digital medicine community. Establishing best practices to address these challenges and employing platforms for digital medicine interoperability will be essential to furthering the field of digital medicine.

## Introduction

Digital medicine is defined as the use of technologies as tools for measurement and intervention in the service of human health [1]. Here we will focus on the use of mobile health (mHealth) and wearable sensors for digital medicine applications, which are growing rapidly in importance for health care applications, particularly during the COVID-19 pandemic. The field of digital medicine has seen rapid growth over the last decade [2]. This growth has resulted from a combination of health care costs and utilization at an all-time high [3] and the consistent improvements in mHealth and wearable technology that have resulted in their wide prevalence and accessibility [4-6].

While the field of digital medicine has seen rapid growth, many challenges remain: standards, best practices, and oversight methodology are still under development [7], sensors and devices used in digital medicine are constantly evolving and are often not validated [8], and a lack of interoperability results in a fragmented, inconvenient, and sometimes impossible adoption of digital medicine into medical practice [9-11]. In order to understand how to mitigate these challenges, it is critical to understand who is using and developing digital medicine tools, which tools are most utilized, and what common perspectives are on current challenges in the field.

Few data are available regarding the landscape of the digital medicine community and the challenges researchers in digital medicine are currently facing. In this study, we surveyed 56 digital medicine professionals to understand the topography of digital medicine, including the background and perspectives of digital medicine professionals, which sensors and file types are being utilized most commonly in digital medicine research, and perspectives on interoperability in digital medicine. The primary objective of this study is to provide information to the digital medicine community that is working to establish frameworks and best practices for interoperability in digital medicine.

## Methods

An open, web-based survey was conducted from May 1, 2020, to July 10, 2020. The survey was conducted using Google Forms, and usability was tested internally prior to survey deployment. The survey consisted of 19 questions over 4 pages. The survey (Multimedia Appendix 1) was disseminated via monthly email newsletter and Slack to the Digital Medicine professional society (DiMe; approximate reach of 1250 digital medicine professionals), via email to 10 subject matter experts who disseminated the survey in their networks, and via social media (Multimedia Appendix 2). A total of 22 tweets and retweets were tweeted on Twitter, deploying the survey to networks totaling more than 1500 individuals. This survey protocol was approved by the Duke University Campus Institutional Review Board (#2020-0450). All participants provided consent and were provided the survey duration and purpose of the study prior to the survey. No personal information was collected or stored. There were no incentives offered to complete the survey. Survey completion after consent was 100%. All respondents (56 participants) were asked questions on background and perspectives on interoperability challenges. If participants checked that they were involved in digital medicine research, research and development (R&D), or both, we asked additional questions on sensors and file types they use (40 participants). Participants were able to review their survey answers prior to submission.

PubMed literature review was conducted on July 14, 2020, with keywords listed in Multimedia Appendix 3. Results were limited to the time span of 2010-2020.

## Results

### Who Makes Up the Field of Digital Medicine?

**Figure 1:**
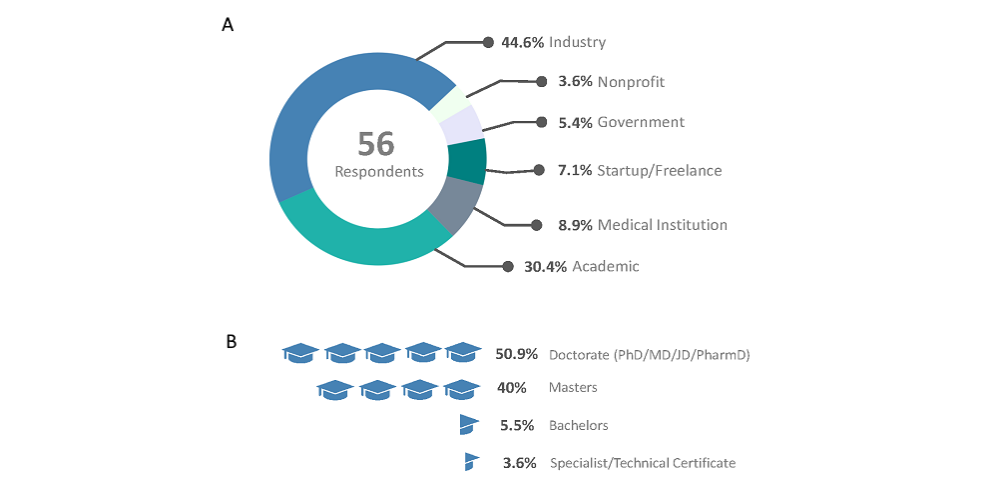
Characteristics of surveyed professionals in the field of digital medicine. (A) Sector breakdown. (B) Terminal degrees.

We found that our sample of the field of digital medicine is made up of people with diverse backgrounds (Figure 1). The most common educational backgrounds and current roles among the 56 survey respondents include data science/analytics/machine learning (18), business/entrepreneurship (17), and medicine (practitioners) (16). However, backgrounds were diverse and included nutrition, psychology, economics, design, marketing, and theatre. The sector breakdown for digital medicine professionals in this survey was industry (25, 45%), academia (17, 30%), medical institution (5, 9%), startup/freelance (4, 7%), government (3, 5%), and nonprofit (2, 4%) (Figure 1A). Just over half (52%, 29) of survey respondents hold a doctorate as their terminal degree, and 39% (22) hold a master’s degree as their terminal degree (Figure 1B).

### Which Sensors Do Digital Medicine Researchers Use?

**Figure 2:**
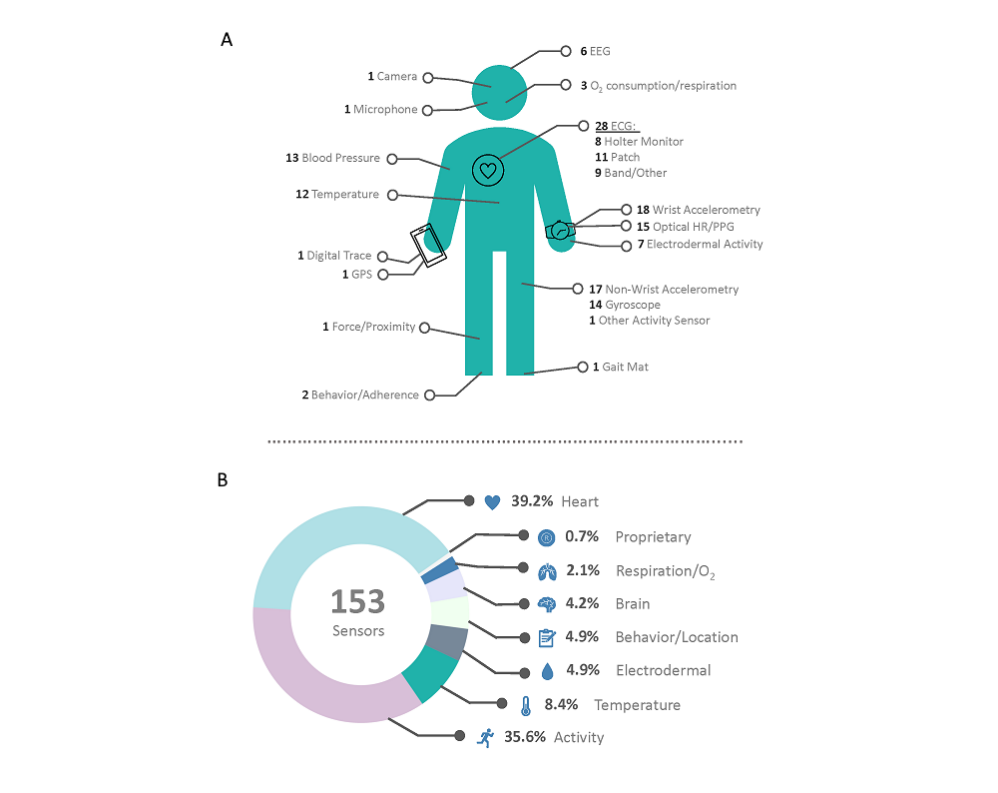
Sensors used by researchers in digital medicine. (A) Types of devices and sensors reported. (B) Measurement modalities of reported devices and sensors. ECG: electrocardiogram; EEG: electroencephalogram; HR: heart rate; PPG: photoplethysmogram.

Respondents who were active participants in digital medicine research and R&D at the time of the survey (n=40) answered with a wide variety of responses to the question “Which sensors and devices do you regularly work with?” A total of 153 sensors were reported to be used by these 40 researchers (mean 3.6, median 3 sensors or devices per respondent; Figure 2). Of these 153 sensors, 143 sensors were associated with a particular measurement modality: 39.2% (56) monitored the cardiovascular system, 35.7% (51) measured physical activity, 8.4% (12) measured physiological temperature, 4.9% (7) measured electrodermal activity, 4.9% (7) monitored behavior, adherence, or location, 4.2% (6) monitored brain activity, 2.1% (3) monitored respiration or oxygen consumption, and 0.7% (1) of sensors were reported as proprietary (Figure 2B). The top three reported devices or sensors used by survey respondents include smartphone (iPhone or Android) (8), Apple Watch (7), and Fitbit (7).

The results of the survey are consistent with the literature: studies indexed in PubMed in the last decade include electrocardiogram (number of articles [n]=59,114), photoplethysmogram (n=2819), accelerometer (n=11,430), electrodermal activity (n=729), temperature sensor (n=11,980), gyroscope (n=1417), and pulse oximetry (n=7638). Smartphones (n=12,485) are also popular tools for medical research. Fitbit was found to be the most common smartwatch cited in PubMed-indexed research (n=624), followed by Garmin (n=141) and Apple Watch (n=136).

### Which Data Formats Are Most Commonly Used in Digital Medicine Research?

**Figure 3:**
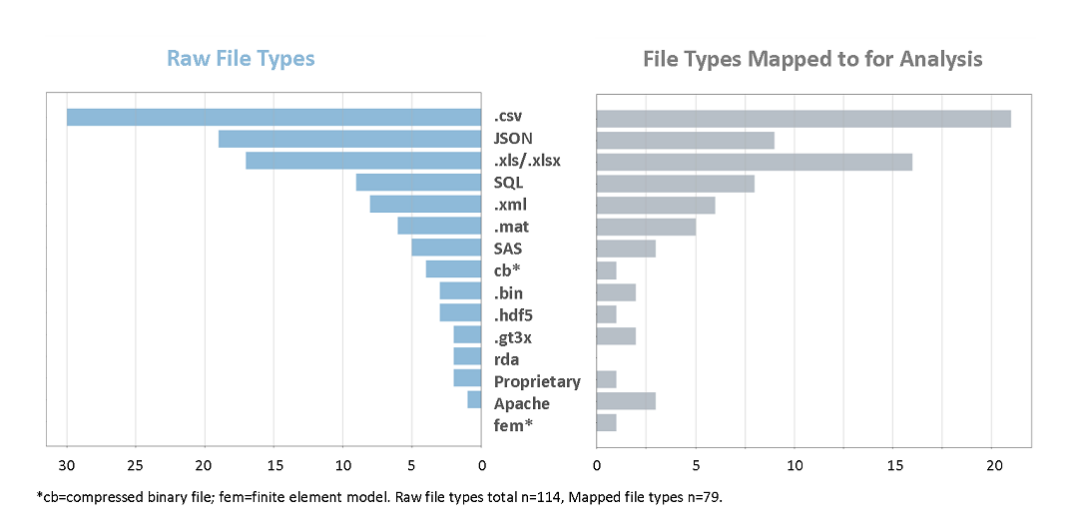
Data formats most commonly used in digital medicine research.

The most commonly used data formats among survey respondents include comma-separated values (.csv), JavaScript Object Notation (JSON), and Microsoft XML spreadsheets (.xls/.xlsx) (Figure 3). The most popular file type for both raw files (data sourced directly from the device or company database) and processed files was .csv. While JSON was used more frequently in raw file types, .xls/.xlsx was more frequently the file type researchers reported to use for analysis (Figure 3). Interestingly, there was more diversity among raw file formats (n=114) than file types that researchers map to for analysis (n=79). For raw file formats, respondents listed a mean of 2.75 file types and a median of 2.5 file types. For analysis file formats, respondents listed a mean of 2 file types and median of 2 file types.

### Interoperability in Digital Medicine

**Figure 4:**
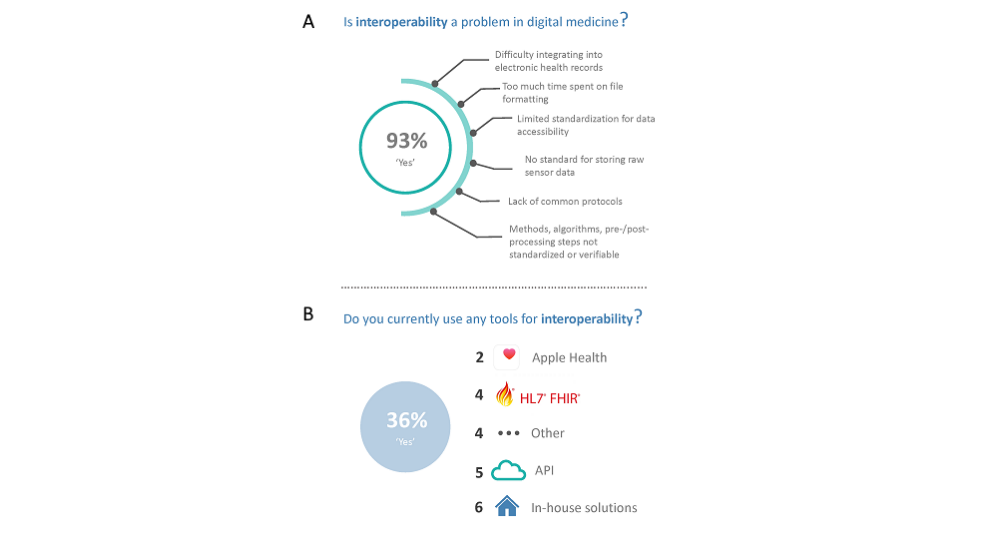
Interoperability in digital medicine. (A) Challenges in digital medicine interoperability. (B) Tools used for interoperability. API: application programming interface; FHIR: Fast Healthcare Interoperability Resources; HL7: Health Level Seven.

Of the 56 survey respondents, 93% (52) agreed that interoperability is a problem in digital medicine (Figure 4A). The most cited challenges in digital medicine interoperability include integration with electronic health records, lack of standard data schemas, and lack of standard and verifiable methods for digital medicine research.

While nearly all respondents (52, 93%) believe interoperability is a problem in digital medicine, only 36% (20) currently use tools for interoperability. Those tools for interoperability include Health Level Seven (HL7) Fast Healthcare Interoperability Resources (FHIR), Apple Health, general application programming interfaces (APIs), the Medisafe platform, Epic, Protege, and in-house solutions (Figure 4B).

When asked if they would utilize a platform for standardizing and validating digital medicine algorithms, methodologies, and analyses, 100% (53) of respondents said “Yes” (27, 51%) or “Maybe” (26, 49%). Reasons for use, as described by survey respondents, included that open science increases efficiency and improves reproducibility, work quality, and readability, and that such a platform would allow for direct comparisons of analytical results. Considerations for using this type of platform included understanding data security and potential risks.

When asked whether they would use a platform mapping raw data files to a standard format, 84% (47) of respondents said “Yes” (13, 23%) or “Maybe” (34, 61%). Considerations for using this platform included understanding compatibility with electronic health record systems, whether this platform would save time, and whether the resulting file types aligned with the desired file types for analysis.

## Discussion

### Overview

In a survey of 56 digital medicine professionals, we sought to better understand the digital medicine community. Key challenges in the field of digital medicine include the following: standards, best practices, and oversight methodology are still under development [7]; sensors and devices used in digital medicine are constantly evolving and are often not validated [8]; and a lack of interoperability discourages adoption of digital medicine into medical practice [9-11]. To understand how these challenges can be mitigated, we explored which sensors are most commonly used in digital medicine research, which file types are most commonly used by the digital medicine community, and perspectives in the field regarding challenges in interoperability.

### Diversity of the Digital Medicine Community Sets Us Apart

We showed great diversity of backgrounds and current roles in the digital medicine community. We hope that this diversity will be encouraging to those looking to join the digital medicine community who may come from “nontraditional” backgrounds. The diversity of backgrounds in the digital medicine community is one of our most important assets for developing a common language, strong frameworks, and best practices. This diversity in thought and experience has resulted in a uniquely heterogeneous set of voices and perspectives contributing to community standards and best practices in research and application [1,7,12-14].

### Digital Medicine Research Is Largely Multimodal

Of the 40 digital medicine researchers in the survey, they regularly work with a total of 153 sensors, indicating that many researchers are using a number of sensors in their research. These sensors are largely used to monitor the cardiovascular system (electrocardiogram, photoplethysmogram, blood pressure) and participant activity (accelerometry, gyroscope, gait map). Literature review of PubMed-indexed studies showed similar results: researchers are primarily employing sensors measuring physical activity and the cardiovascular system in their work. Many are also using temperature, electrodermal activity, or electroencephalogram sensors in their research. Overall, smartphones continue to be the dominant source of digital health information collection in the digital medicine community.

### There Is Not a Consensus on File Types in Digital Medicine

In order to inform teams working to establish common data schemas and file types, we examined file types that are commonly used in digital medicine. There are 15 unique file types used by digital medicine researchers, either as raw files or as file types mapped to for analysis. While the .csv file type is the most commonly used, there are other commonly used file types, including JSON and .xls/.xlsx. There was more diversity among raw file formats (n=114) than file types researchers map data to for analysis (n=79), and respondents are averaging 2.75 raw file types versus a mean of 2 file types for analysis, indicating that while researchers may receive raw data in a number of file formats, they are mapping them to a smaller subset of file types for analysis. We show that there is not a general consensus on file types in digital medicine and data is currently handled in multiple ways (both as raw files and as files mapped for analysis). Noteworthy is the low number of proprietary file types, indicating that the digital medicine community is largely using accessible file types that could be mapped to a standard, interoperable format.

### Interoperability Remains a Critical Challenge in Digital Medicine

Nearly all digital medicine professionals surveyed (52/56, 93%) agree that interoperability is a problem in digital medicine. Literature points to this lack of interoperability being a critical barrier to using digital medicine in clinical practice, causing fragmented, inconvenient, and sometimes impossible clinical adoption of digital medicine [9-11]. While many specific challenges to digital medicine interoperability were revealed in this study, the most cited challenges include integration with electronic health records, lack of standard data schemas, and a lack of standard and verifiable methods for digital medicine research. These three areas should be the focus of future directions in developing standards, frameworks, and best practices for interoperability in digital medicine.

Despite agreeing that interoperability is a problem facing the digital medicine community, only 36% (20) of respondents currently use existing tools for interoperability. Generally employed tools used in the community include HL7 FHIR, Apple Health, unspecified APIs, and in-house solutions. A large proportion of those declaring that they use interoperability tools use in-house solutions, which addresses a key problem in the current field of digital medicine—siloed solutions that are not generalizable and are adopted by only a small number of digital medicine professionals.

One of the critical interoperability needs identified is the development of standard data schemas. When asked whether they would use a platform mapping raw data files to a standard format, respondents identified considerations that would have to be made to use this platform: they would need to understand compatibility with electronic health record systems and whether this platform would save time. The most cited consideration for using a platform mapping raw data files is the standard format that data would be mapped to. We identified that there is a strong preference for .csv and .xls/.xlsx filetypes for data analysis among respondents. When developing standard data schemas for the field of digital medicine, it is important to consider the most commonly used file formats and how standard formats could map from and between these popular file formats. Open mHealth, currently the leading mobile health data interoperability standard, maps data to a common JSON data structure [15].

Other critical interoperability needs included standard and verifiable methods for digital medicine research, including preprocessing and postprocessing, algorithms, models, and analyses. When asked if they would use a platform for standardizing and validating digital medicine algorithms, methodologies, and analyses, respondents identified considerations for using this platform, which include understanding data security, potential risks, and extensibility. MD2K Cerebral Cortex provides a complete software platform that allows for data collection and analysis with interactive web dashboards [16]. Recently, we identified a need for an open-source, crowdsourced software platform where digital medicine researchers could share and compare methods, algorithms, and processing methods. To address this need, we have developed the Digital Biomarker Discovery Pipeline (DBDP) [3]. Future directions for the digital medicine community include addressing and implementing solutions to the critical interoperability needs in digital medicine: integration with electronic health records, standard data schemas, and standard and verifiable methods for digital medicine research.

This study was limited in the small sample size and the short time frame of the survey; thus, extending this work will be necessary to further understanding of the challenges facing the digital medicine community as research in mHealth and wearables expands.

In conclusion, understanding the digital medicine community, the sensors and file types commonly used, and perspectives on interoperability will enable the development and implementation of solutions to the critical interoperability needs in digital medicine. As the digital medicine community builds tools, platforms, and resources for mobile health and wearable sensor data, this study can be leveraged to meet real needs and address existing technology gaps. The challenges to interoperability outlined by this study will drive the next steps in creating an interoperable digital medicine community. Establishing best practices to address these challenges and employing platforms for digital medicine interoperability will be essential to furthering the field of digital medicine.

## Appendix

Multimedia Appendix 1 Web-based survey.

Multimedia Appendix 2 Recruitment blurb for distribution of survey via email, Slack, and social media.

Multimedia Appendix 3 PubMed literature review was conducted on July 14, 2020, with the following keywords. Results were limited to the time span 2010-2020 to account for newer technologies.

## Abbreviations

API: application programming interface
.csv: comma-separated values
DiMe: Digital Medicine professional society
FHIR: Fast Healthcare Interoperability Resources
HL7: Health Level Seven
JSON: JavaScript Object Notation
mHealth: mobile health
R&D: research and development
.xls/.xlsx: Microsoft XML spreadsheets
