# Supplementary material for: Digital Medicine Community Perspectives and Challenges: Survey Study
Source: JMIR Mhealth Uhealth. 2021 Feb 3;9(2):e24570. doi: 10.2196/24570 (PMC7889423; doi:10.2196/24570)
Supplement: Multimedia Appendix 1 [file mhealth_v9i2e24570_app1.pdf]

Digital Medicine Landscape and Interoperability Questionnaire

This is a survey developed by the Big Ideas Lab at Duke University to survey the digital health community about software interoperability and determine the current landscape of digital medicine.

* Required

Consent

Key Information
The purpose of this research is to understand the landscape of digital medicine, what tools are being used for digital medicine, and what challenges are facing the community.

The questionnaire will take approximately 10 minutes to complete. We do not collect any personal identifying information in this survey. Your participation in this survey is voluntary and you can opt out of the survey at any time and your response will not be recorded. This study does not have any risks associated with it.

By clicking 'Next', you are consenting to allow us to use your responses in future and current research, publications, reports, and/or marketing materials.

This questionnaire is being conducted by the Big Ideas Lab at Duke University. Please contact the research team with any questions or comments at jessilyn.dunn@duke.edu or brinnae.bent@duke.edu. For questions about your rights as a participant in this research study, contact the Duke University Campus IRB at 919-684-3030 or campusirb@duke.edu.

Protocol ID#: 2020-0450

Background Questions

We know the community of digital medicine is made up of individuals with a variety of backgrounds. We want to understand and highlight this diversity!

1. What is your highest level of education?

Mark only one oval.

- Bachelors
- Masters
- Doctorate (PhD, MD, JD, PharmD, etc)
- High School or GED
- Other: ______________________

2. Please select the option below that best matches your current role. *

Mark only one oval.

- Industry
- Academic
- Government
- Medical Institution
- Other: ______________________

3. Please select the option below that best matches your current role and/or education: *

Check all that apply.

- [ ] Engineering
- [ ] Medicine
- [ ] Business/Entrepreneurship
- [ ] Law
- [ ] Education
- [ ] Statistics/Biostatistics
- [ ] Data Science/Analytics/Machine Learning

Other: [ ] ____________________

4. What is your current role (i.e. what do you do?) *

____

5. Do you work in digital medicine research/ R&D? *

Mark only one oval.

( ) Yes *Skip to question 6*

( ) No *Skip to question 10*

Software/Hardware Questions

6. What sensors/devices do you regularly work with? *

Check all that apply.

- [ ] Accelerometry/actigraphy (wrist wearable)
- [ ] Accelerometry/actigraphy (non-wrist sensor)
- [ ] PPG/optical heart rate sensor
- [ ] Electrocardiogram, ECG (Holter Monitor)
- [ ] Electrocardiogram, ECG (Patch)
- [ ] Electrocardiogram, ECG (Band, other)
- [ ] Electroencephelography, EEG
- [ ] Electrodermal Activity/Galvanic Skin Response
- [ ] Temperature
- [ ] Gyroscope
- [ ] Blood pressure
- [ ] None/I do not work with sensors/devices

Other: [ ] ______________________

7. What devices/sensors do you use in your research? (Please be specific and give make and model when applicable). If you use in-house developed sensors or devices, please add as many details as you can. (i.e.The HR sensor on a Fitbit) *

____

____

____

____

____

8. What raw file type(s) do you use in your analyses? *

Check all that apply.

- [ ] .csv
- [ ] .xls/.xlsx
- [ ] .mat
- [ ] .hdf5
- [ ] JSON
- [ ] .gt3x
- [ ] .bin
- [ ] SAS
- [ ] SQL
- [ ] rda
- [ ] Apache
- [ ] Compressed binary files
- [ ] .xml

Other: [ ] ____________________

9. What file type(s) do you map your raw files to for analyses? *

Check all that apply.

- [ ] .csv
- [ ] .xls/.xlsx
- [ ] .mat
- [ ] .hdf5
- [ ] JSON
- [ ] .gt3x
- [ ] .bin
- [ ] SAS
- [ ] SQL
- [ ] rda
- [ ] Apache
- [ ] Compressed binary files
- [ ] .xml

Other: [ ] ____________________

Interoperability Questions

10. Do you think interoperability is a problem in digital medicine? *

Mark only one oval.

- ( ) Yes
- ( ) No

11. What are some problems with interoperability in the field of digital medicine?

12. Do you currently use any tools for interoperability in digital medicine? *

Mark only one oval.

- Yes
- No

13. If yes, what tools do you currently use?

14. Have you heard of Open mHealth?

Mark only one oval.

- Yes
- No
- Maybe

15. Have you used Open mHealth Shimmer to map mobile health data to JSON?

Mark only one oval.

- Yes
- No

16. Would you utilize a platform (similar to Open mHealth) that maps raw data files to a standard format (i.e. JSON)?

Mark only one oval.

- Yes
- No
- Maybe

17. Why did you choose your answer for the previous question?
