# Supplementary material for: Digital Medicine Community Perspectives and Challenges: Survey Study
Source: JMIR Mhealth Uhealth. 2021 Feb 3;9(2):e24570. doi: 10.2196/24570 (PMC7889423; doi:10.2196/24570)
Supplement: Multimedia Appendix 2 [file mhealth_v9i2e24570_app2.pdf]

**Recruiting blurb for Digital Medicine Society weekly emails and Slack*:**
Calling all digital medicine stakeholders, researchers, clinicians, and professionals. We are trying to understand the landscape of digital medicine, what tools are being used for digital medicine, and what challenges are facing the community. Please fill out this brief questionnaire about your role in digital medicine, what tools you utilize, and what challenges you think the community is facing. <<Link to survey here>>

(*For social media posts with character limits, this recruiting blurb was shortened and potentially re-arranged)
