# Supplementary material for: Digital Medicine Community Perspectives and Challenges: Survey Study
Source: JMIR Mhealth Uhealth. 2021 Feb 3;9(2):e24570. doi: 10.2196/24570 (PMC7889423; doi:10.2196/24570)
Supplement: Multimedia Appendix 3 [file mhealth_v9i2e24570_app3.docx]

**Supplementary Table S1.** PubMed literature review was conducted on July 14, 2020 with the following keywords. Results were limited to the time span 2010-2020 to account for newer technologies.

| **Keyword(s)** | **Results 2010-2020** |
| --- | --- |
| digital + medicine | 22564 |
| digital + health | 22096 |
| digital + biomarker | 3994 |
| wearable + medicine | 2940 |
| wearables | 12619 |
| sensor + medicine | 15574 |
| mobile + health | 43892 |
| mobile + medicine | 19693 |
| mHealth | 26907 |
| smartwatch | 321 |
| smartphone | 12485 |
| Fitbit | 624 |
| Apple + Watch | 136 |
| Garmin | 141 |
| iPhone | 886 |
| accelerometer | 11430 |
| ECG | 59114 |
| PPG | 2819 |
| photoplethysmography | 1930 |
| electrodermal + activity | 729 |
| temperature + sensor | 11980 |
| gyroscope | 1417 |
| pulse + oximetry | 7638 |
